# Comparing the Fiscal Consequences of Controlled and Uncontrolled Osteoarthritis Pain Applying a UK Public Economic Perspective

**DOI:** 10.36469/001c.24629

**Published:** 2021-06-28

**Authors:** Rui Martins, Nikos Kotsopoulos, Melodi Kosaner Kließ, Craig Beck, Lucy Abraham, Samuel Large, Patricia Schepman, Mark P. Connolly

**Affiliations:** 1 Health Economics, Global Market Access Solutions; 2 Health Economics, Global Market Access Solutions; Economics, University of Athens; 3 Pfizer Inc.; 4 Health Economics, Pfizer Inc.; 5 Health Economics, Global Market Access Solutions; Pharmacoeconomics, University of Groningen

**Keywords:** controlled pain, public economics, fiscal analysis, economic evaluation, osteoarthritis pain, osteoarthritis

## Abstract

**Background:** Individuals experiencing osteoarthritis (OA) pain can pose significant costs for governments due to reduced work activity in these individuals and increasing reliance on public support benefits. In this analysis we capture the broader economic impact of OA pain by applying a government perspective, public economic framework to assess controlled and uncontrolled pain.

**Methods:** We used a Markov model to compare labour market participation in people with uncontrolled OA hip or knee pain compared to a cohort with controlled OA pain. The likelihood of employment, long-term sickness, disability, and early retirement in those with controlled pain used publicly available UK data. The relative effect of uncontrolled OA pain on fiscal outcomes is drawn from peer reviewed publications reporting reduced work activity and reliance on public benefits for people with uncontrolled OA pain. Lost tax revenue was derived using UK tax rates and national insurance contributions applied to annual earnings. Social benefit rules were applied to calculate government financial support to individuals. Health-care costs were calculated based on estimates from an UK observational study. The base case analysis compared the projected lost tax revenue and transfer payments for a 50-year-old cohort with severe OA pain, retiring at age 65.

**Results:** For a 50-year-old individual with moderate uncontrolled OA pain with 15-years remaining work expectancy, the model estimated a £62 383 reduction in employment earnings, a £24 307 reduction in tax contributions and a need for £16 034 in government benefits, compared to a person with controlled OA pain. In people with severe uncontrolled OA pain incremental foregone earnings were estimated to be £126 384, £44 925 were not paid through taxation and £25 829 were received in public benefits, compared to the controlled pain cohort. Health-care costs represented 13% and 12% of all OA-related fiscal cost in the moderate uncontrolled OA pain and severe uncontrolled OA pain comparison, respectively.

**Conclusions:** For governments, maintaining an active workforce is paramount to maintaining economic growth and reducing spending on government programs. The approach described here can be used to augment cost-effectiveness models to inform a range of stakeholders of benefits attributed to controlled OA pain.

## INTRODUCTION

Osteoarthritis (OA) is the most common form of arthritis affecting 10-12% of the adult population and the 11th most debilitating disease worldwide accounting for over 17 million years lived with disability.^1–3^ It affects highly utilized joints in the body, more frequently in women.^4,5^ Commonly, OA manifests from the age of 40-50, but incidence has been increasing in younger individuals.^6,7^ The highest burden comes from mobility-related disability due to hip and knee progressive pain and stiffness, often leading to joint replacement in later stages of the disease.^8,9^ Walking disability and OA have in turn been shown to correlate with increased mortality.^10,11^ In 2017, global age-standardised prevalence and incidence of OA were estimated at 3754.2 and 181.2 per 100 000 population, a 9.3% and 8.2% increase from 1990, respectively.^9^ In the United Kingdom, where OA and low-back pain are the main contributors to chronic pain, the incidence of knee OA rose by 2.9% and hip OA by 3.8% between 2000-2018 with over 3 million people presenting with the disease.^6^ Approximately one third of people aged 45 or more, a total of 8.75 million, have sought treatment for OA in the United Kingdom.^12^

Ageing populations, longer life-expectancy, obesity, and absence of disease modifying drugs are likely contributory factors to the upward trends observed.^4,8^ The clinical and economic burden of OA is substantial, with costs accounting for 0.5% of a typical developed country’s gross domestic product.^13^ For individuals experiencing OA pain, the condition fits in a biopsychosocial framework marked by persistent bodily pain, fatigue, reduced self-efficacy, loss of independence, leading to decreased social participation and quality of life comparable to those of cardiovascular disease and cancer.^3^ To the labour markets, uncontrolled OA pain leads to more working-age individuals being unable to maintain regular employment,^14^ increased absence due to sickness and increased inactivity due to disability or early retirement.^15,16^ All these factors can pose costs for government in the form of increased disability payments and lost tax revenue attributed to reduced work activity in these individuals.^17,18^ In this study, we quantified how uncontrolled OA pain affects public economics for the UK government by estimating gross tax losses and costs from social benefits (transfers) compared to people with controlled OA pain. This was done using a generational accounting framework that considered government tax revenue and social benefits transfers paid to individuals with OA pain, in addition to health-care costs.^19,20^ The analysis described here will help decision-makers better understand the broader impact of OA attributable pain, and the likely benefits in the form of increased tax revenue and reduced benefits transfers associated with reducing uncontrolled OA pain.

## METHODS

### Model Structure

The model was developed as a comparative analysis using a public economic^20,21^ perspective of the government specific costs. A Markov-chain structure with annual cycles was used to simulate the fiscal pathway of a person with OA entering the model at age 50 and the impact of uncontrolled pain due to OA on labour force participation and need for government social benefits. We defined people with a Western Ontario and McMaster Universities Osteoarthritis Index (WOMAC) score (range 0 to 100) below 7 would have controlled pain (asymptomatic) and would have a similar labour market participation to individuals in the general population. People with WOMAC scores above 7 were deemed to have uncontrolled pain.^14,22^ The base case used an average remaining work expectancy of 15 years. Lifetime results were also presented using a scenario analysis. The proportion of females (58.6%) was calculated from a publication estimating OA incidence trends using a large sample of UK patients in secondary care.^6^ The basic structure of the model is depicted in a diagram included in the **Supplemental Materials**.

### Baseline Probabilities

At the start of the simulation, the cohort distribution mimicked that of the general UK population. The distribution of employment, unemployment, long-term sickness (LT sickness), disability, and early retirement fiscal states was sourced from datasets published by the Office of National Statistics (Table 1). Employed and unemployed people were considered active, those on LT sickness and retiring early were deemed inactive. Baseline probabilities of being active were gender-specific to account for the higher prevalence of OA in females and allowed separate results for males and females. The rates of LT sickness and early retirement were reported as single means, and as proportions of the total inactive population.^23^ For this reason, we calculated age-stratified probabilities of transitioning to LT sickness and early retirement by multiplying the reported means by the proportion of the cohort not employed or unemployed at each age category. The probability of disability was calculated using the age-adjusted probability of being disabled,^24^ and the age-adjusted prevalence of economic inactivity for people with disabilities up to the age of 65.^25^ People were not allowed to transition to the unemployment or early retirement state after reaching the UK state pension age (SPA, 65 years).^26^ Individuals could still be employed beyond SPA, but it was assumed that 70 would be the maximum working age in the model. Unemployment and LT sickness were implemented as tunnel states to address the lack of “memory” in Markov models. People who remained alive and did not transition to any of the fiscal states were assumed to be tax neutral, remaining in the unknown fiscal consequences state for the entire cycle and being sent to the employment state in the following cycle. It was assumed the people could return to employment after being unemployed or on LT sickness but not after disability or early retirement. It was assumed that people with controlled OA pain would demonstrate labour participation patterns similar to those of the general population, whilst people with uncontrolled pain would be subject to OA pain-related limitations impacting their ability to maintain employment and normal retirement age. The scale of these limitations was informed by publications identified through a targeted literature review conducted for the purpose of the analysis.

**Table 1.** Baseline Rate and Fiscal States Probabilities in People with Controlled OA Pain*

Age groups	Employment^†^	Unemployment^†^	LT Sickness	Early Retirement	Disability
50 to 54	0.718	0.028	0.031	0.017	0.039
55 to 59					0.043
60 to 64					0.059
65 to 69	0.107	0.016	0.061	0.033	0.369
70 to 74	0.015	0^‡^	0^‡^	0^‡^	0^‡^
75 to 79	0^‡^				
80 to 84					
85 to 89					
90 to 90+					
Source	^27^		^23^		^24,25^

*Probabilities do not add to 1 as some people with OA will fall into the state of unknown fiscal consequences.

†Weighted average from split gender results using a 41.4% OA prevalence in males, data covering the period from May-July 2018 to May- July 2020.

‡Model assumptions.

### Targeted Literature Review

To inform the limitations imposed by OA pain, a targeted literature search of PubMed, Embase, EconLit and CINHAL were performed in May 2020 using a strategy composed of search terms specific to hip and knee OA pain and public economics. Results were limited to observational studies conducted in adults, published in English language since 2010. Relevant systematic reviews were manually checked and keyword internet searches were performed with the aim of identifying additional references. The resulting titles and abstracts were sifted by a single reviewer using prespecified inclusion criterion. Publications deemed relevant for full text inspection were independently scanned by two experienced reviewers. Inclusion disagreements were resolved by discussion. Publications from countries with social welfare systems identical to the United Kingdom’s and reporting on the relative likelihood of fiscal outcomes were finally selected to inform the economic model. The PubMed search strategy and the review CONSORT diagram can be found in the **Supplemental Materials**.

### Relative Measures of Effect

**Figure 1.**
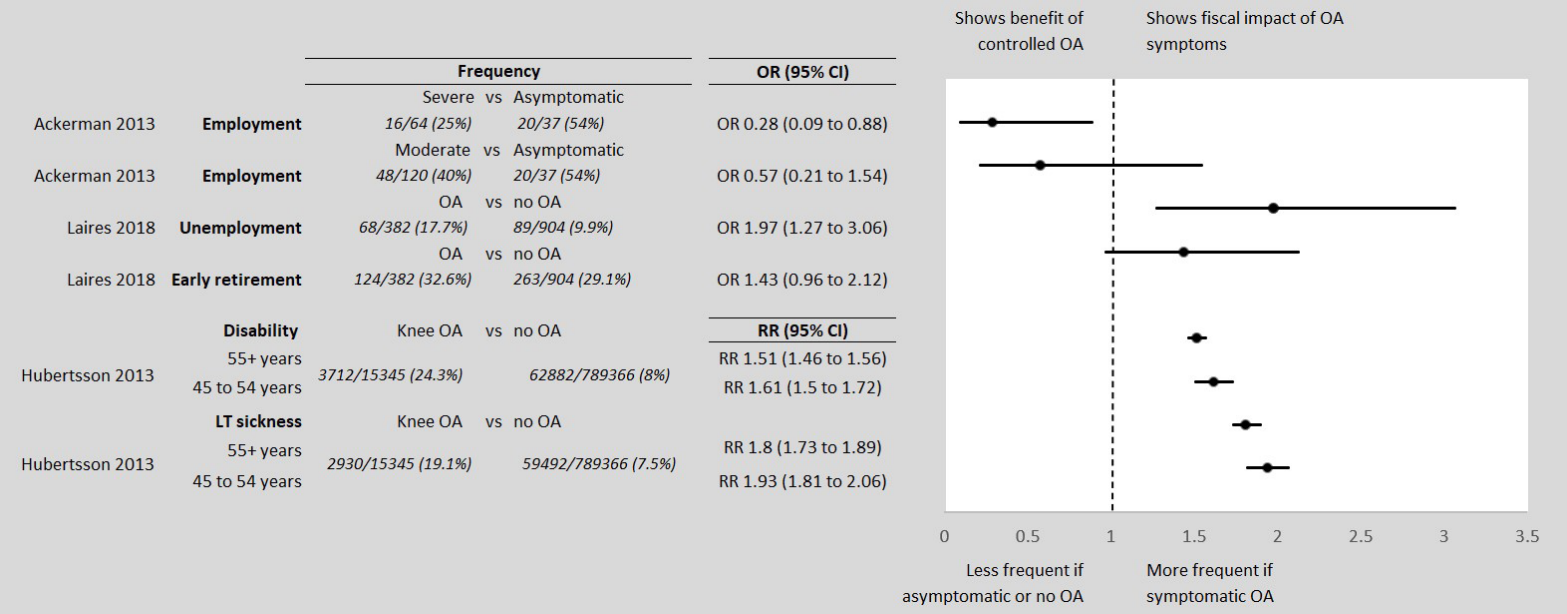
Direction of Effect of the Relative Measures of OA Fiscal Burden Abbreviations: CI, confidence interval; LT, long-term; OA, osteoarthritis; OR, odds ratio, RR, relative risk.

The publications identified in the targeted literature review suggest that people with uncontrolled OA pain are less likely to be employed than those who are asymptomatic,^14,16^ and more likely to be on LT sick leave,^15^ to have a disability,^15^ and retire earlier^16^ than people with no OA attributable pain. These measures of additional fiscal burden, reported as odds or risk ratios in the literature, were applied to the baseline fiscal states probabilities to generate the state transition in the uncontrolled pain cohort. The direction of effect and significance level of the estimates are plotted in Figure 1. Ackerman and colleagues reported different likelihoods of employment for people with moderate and severe joint disease severities due to OA. Severity was defined using a WOMAC score of 7 to 38 for mild to moderate joint disease due to OA and ≥39 for severe,^14^ a previously utilized classification.^22^ In the model we assumed joint disease severity due to OA to be a proxy for controlled/uncontrolled OA pain. Hubertsson et al.^15^ reported age-specific relative risks (RR) of sick leave not covered by employers and receiving a disability pension in those with knee OA versus no OA. Laires et al.^16^ reported odds ratios (OR) for unemployment and early retirement in people with OA of the hip or knee compared to a cohort without OA. We assumed that people with controlled OA pain would have a labour market participation identical to that of people without OA. People with an indication of total joint replacement are likely to have decreased labour participation for a sustained period, perioperatively. Due to surgery efficacy, possible complications, and age some people will be more likely to return to work and others will be less likely to return to work.^28–32^ In the interest of simplicity, we did not model the effect of joint replacement surgery on labour force participation.

### Death

Death was possible from all fiscal states using rates from UK lifetables^33^ and accounted for a higher proportion of females in the model.^6^ Excess OA mortality was sourced from a UK population-based prospective cohort study conducted in adults over the age of 50, hazard ratio (HR) 1.11, 95% confidence interval (CI) 1.06 to 1.17.^34^ The base case analysis did not consider mortality to be a differential between people with controlled and uncontrolled OA pain but a scenario was modelled using data from a Canadian prospective cohort suggesting that a 10-point increase in the WOMAC score was associated with an increased risk of death, HR 1.04, 95% CI 1.01 to 1.07.^11^ When required, rates were converted to cycle length probabilities using **Equation 1**.

(1)p=1−exp(ln⁡(1−pi)ti∗t)

where *p* is the index probability, *t* the index time period, and *t* is the cycle length.^35^

When the outcomes were not rare (<10%), OR were converted to RR using **Equation 2**.

(2)RR=OR(1−p0+(p0×OR))

where *p_0_* is the probability of the event in the unexposed.^36^

### Fiscal Consequences

Each fiscal state had one or more monetary consequences in the form of taxation, transfers to individuals or health-care costs. These values were multiplied by the annual probability of occupying a fiscal state to calculate its annual cost. Direct taxes were obtained by multiplying the UK age-stratified median income^37^ by the 30.9% tax wedge paid by employees and employers.^38^ Indirect taxation on disposable income was calculated using a 13.7% rate applied to labour income or to the total income from state benefits.^39^ People under SPA who were unemployed were entitled to a jobseeker’s allowance.^40^ We accounted for the costs of LT sickness (employment and support allowance) only as the first 28 weeks of statutory sick pay, which are usually covered by the employer.^41^ People with disability and below SPA were allowed a personal independence payment.^42^ Finally, a basic state pension was assigned to those retiring earlier or reaching SPA.^43^ After retirement, half of those classed as disabled were assumed to have severe dependency and were given attendance allowance in addition to their basic state pension.^44^ Allowances were often sourced as weekly wages; Table 2 shows the annualised taxes and transfers utilized in the model.

**Table 2.** Annualised Tax and Transfers (Per capita)

	**Age Groups**			**Source**
Fiscal Consequence	50 to 59	60 to 64	65+	
Tax on Labour Income^±^	£10 886	£10 015	£8825	^37,38^
Indirect Tax	13.7% of gross income			^39^
Jobseeker’s Allowance	£3866	NA	^40^	
Employment and Support Allowance^†^	£4885			^41^
Personal Independence Payment^‡^	£6102			^42^
Basic State Pension^§^	£9110			^43^
Attendance Allowance*			£3258	^44^

Abbreviations: NA, not applicable.

^±^Calculated by multiplying age specific earnings by the UK tax wedge.

^†^Calculated as a weighted average assuming 50% of people would be in the work-related activity group and 50% would be in the support group.

^‡^Calculated using an average of the daily living and mobility benefits allowances reported as minimum and maximum at GOV.UK.

^§^Same for people retiring early and for people achieving state pension ag.

*Assumed 90% would require the minimum attendance allowance and 10% would require the maximum.

### Health-care Costs

Health-care cost estimation was based on the units and frequency of utilization reported by a retrospective observational study conducted in five general practices in Scotland, England, Northern Ireland, and Wales.^45^ The publication specifies the class and frequency of drug use, referrals, appointments, and admissions required in the management of chronic pain in people with a recent or established diagnosis of OA or low back pain. These were combined with unitary costs of each resource extracted from standard national publications to generate the total costs of health care in those with controlled pain. We assumed that the cohort reported by Hart and colleagues represented an average of the typical OA pain management pathway and that people with uncontrolled OA pain would require 20% or 30% additional resources to manage their symptoms of uncontrolled moderate or severe OA pain, respectively. In sensitivity analysis, we varied the incremental cost difference using plausible ranges to assess the impact of health-care costs on the model results. The costs of surgery were calculated as the weighted average of major hip/knee procedures for non-trauma.^46^ The rate and costs of aseptic and septic surgical revision were extracted from published UK studies. Table 3 summarizes the health-care costs, more detailed costing information is available in the **Supplemental Materials**.

**Table 3.** Utilization and Costs of Health-care Resources

	Annual % Patients	Source	Annual Cost	Source
Drug Treatment for Pain				
Non-opioid	86.74%	^45^	£64	^47^
Opioid	96.21%		£125	
Adjuvant Analgesic Drugs	58.33%		£13	
Referrals for Pain Management	70.82%		£82	^46,48^
Appointments	^†^		£631	
Surgery				
Primary Hip Surgery	^†^	^49^	£6714	^46^
Primary Knee Surgery	^†^		£6296	
Surgical Revision Hip (aseptic)	0.47%	^50^	£12 444	^51^
Surgical Revision Hip (septic)	0.04%		£22 946	
Surgical Revision Knee (aseptic)	0.35%		£10 325	^52^
Surgical Revision Knee (septic)	0.09%		£32 094	
Annual Cost of Drugs	£203			
Annual Cost of Appointments^‡^	£713			

^†^Multiple parameters, reported in the online supplement data.

^‡^Assumed to be 30% higher in people with uncontrolled OA pain.

### Model Results

Fiscal transfers were deemed “negative” costs to represent government expenditures, as opposed to revenue from taxation. The fiscal monetary difference between people with controlled and uncontrolled OA pain was expressed as an incremental net tax (INT), calculated by subtracting the net present value (NPV) for each cohort. The NPV was derived using the equations below.

(3)NPV=∑t0tTaxt−Costt(1+r)t

where *r* is the annual discount rate of 3.5%^53^ and *t* is time,

(4)Taxt=Directtaxt+Indirecttaxt+Nationalinsurancet

(5)Costt=Healtht+Transferst+Oldagepensiont

### Sensitivity Analysis

One-way sensitivity analyses (OSA) were conducted using the lower and upper bounds of the CIs for the parameters sourced from peer reviewed publications (relative measures of effect).^14–16^ We did not subject baseline probabilities to these analyses as they were sourced from nationwide data. Similarly, fiscal states costs are nationally available tariffs kept relatively stable over time and thought not to be the source of uncertainty in the model.

Scenario analyses were also performed to explore plausible variations of key model assumptions such as age at disease onset, time horizon, age of retirement, and incremental health-care costs.

We implemented a probabilistic sensitivity analysis (PSA) to assess the impact of parameter uncertainty on the results of the model. Commonly utilized distributions were fitted to the model inputs and used to produce 10 000 random samples.^35^ Because we are not assessing the impact of a technology, it is not meaningful to use the UK willingness to pay threshold to express the PSA results. Consequently, we report the average INT and 95% credible intervals resulting from the 10 000 PSA simulations.

## RESULTS

Model results are reported as incremental differences in total taxes and transfers and are discounted at 3.5% annually.

### Base Case

The base case disaggregated taxes and costs are shown in Table 4. For a 50-year-old individual with moderate uncontrolled OA pain the model estimated £69 383 in lost earnings leading to a 30% reduction in total paid taxes (£24 307) compared to controlled OA pain over a 15-year work expectancy. Additionally, an individual with moderate uncontrolled OA pain would receive an excess of £16 034 in social benefits programs compared to those having controlled OA pain. Health-care costs represented 12.7% of overall government costs. Over a 15-year period, uncontrolled OA pain is believed to originate a loss of £40 341 per capita to public finances. Over the same time horizon, for an individual with severe uncontrolled OA pain, we estimated £126 384 in lost earnings leading to £44 925 in reduced tax contributions compared to an identical individual with controlled OA pain. Health-care costs added to 11.8% of the total transfers provided by governments. The resulting INT was £70 754, close to double the amount from the moderate pain scenario.

**Table 4.** Disaggregated Base Case Results for People with Uncontrolled Moderate or Severe OA Pain Compared to Controlled OA Pain

	**Controlled OA Pain**	**Uncontrolled Moderate OA Pain**	**Incremental**	**Uncontrolled Severe OA Pain**	**Incremental**
Earnings	£208 968	£139 586	£69 383	£82 584	£126 384
Gross Tax Revenue	£82 172	£57 865	£24 307	£37 247	£44 925
Job-Seeking Allowance	£1002	£1592	-£589	£1335	-£332
Employment and Support Allowance	£2001	£4280	-£2279	£5476	-£3475
Personal Independence Payment	£6933	£14 005	-£7072	£18 657	-£11 724
Early Retirement Pension	£5727	£9786	-£4059	£12 973	-£7246
Basic State Pension + Attendance Allowance	£0	£0	£0	£0	£0
Health-care costs	£10 390	£12 424	-£2034	£13 441	-£3051
Total Transfers	£26 053	£42 087	-£16 034	£51 882	-£25 829
Net Tax	£56 119	£15 778	£40 341	-£14 634	£70 754
Life-Years	11.101	11.101	0.000 *	11.101	0.000 *

Abbreviations: OA, osteoarthritis.

*In the base case, pain was deemed not to affect mortality differentially.

### Sensitivity Analysis

**Figure 2.**
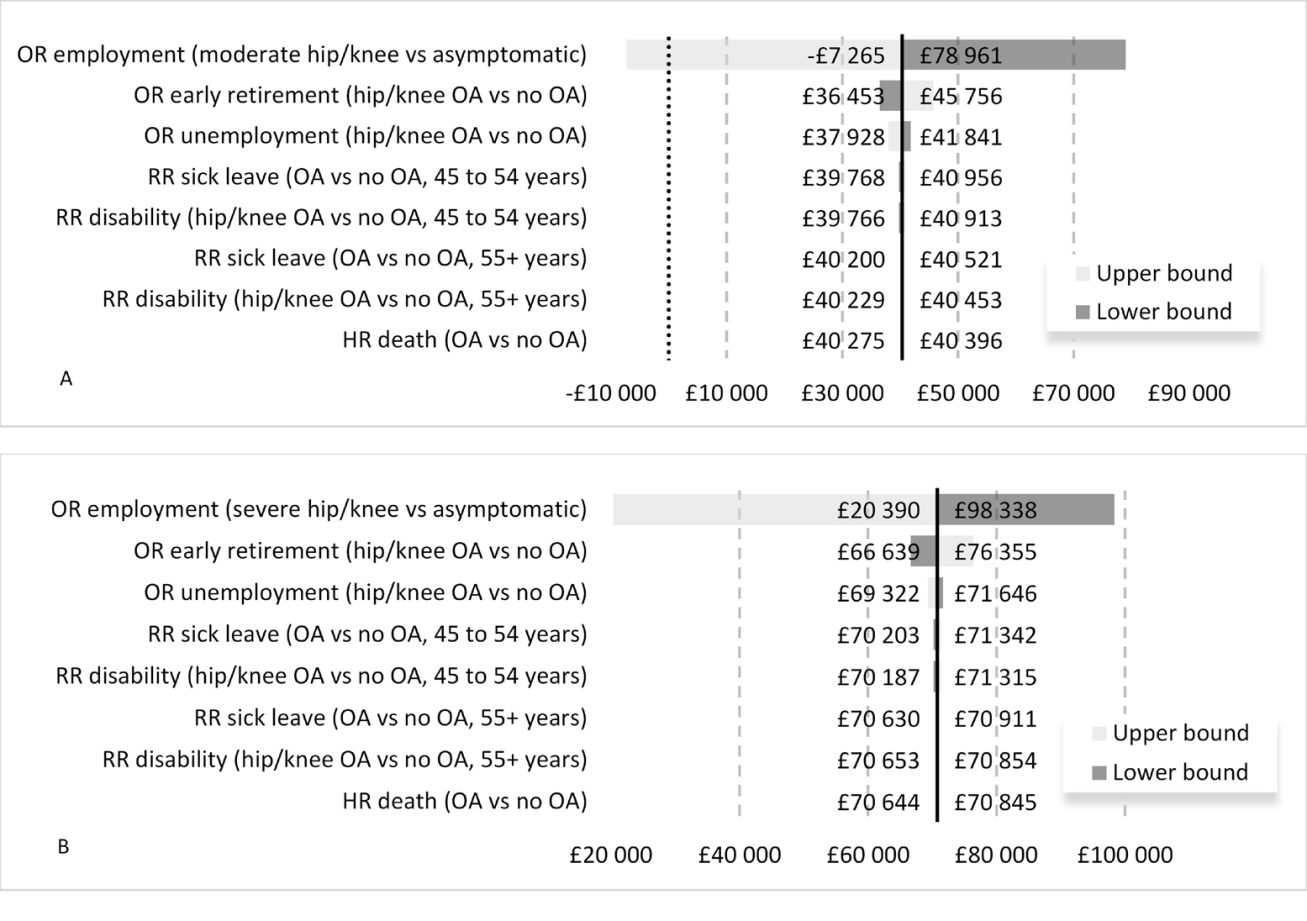
Tornado Diagram A - Uncontrolled moderate OA pain scenario, B - Uncontrolled severe OA pain scenario. Abbreviations: HR, hazard ratio; OA, osteoarthritis; OR, odds ratio; RR, relative risk.

In OSA the ORs of employment in people with moderate and severe OA pain were the only parameters significantly influencing the results in the model Figure 2. The remaining parameters caused the INT value to vary by a maximum of 20% in the moderate pain scenario and 13% in the severe pain scenario and were not sufficient to change the conclusions of the analysis.

At the upper end of the 95% CI the OR of employment in people with moderate uncontrolled OA pain was 1.54. Counterintuitively, this would imply that people with moderate uncontrolled pain were more likely to be employed than identical individuals with controlled OA pain. The resulting net tax difference was -£7265. The mean estimates of the PSA were similar to the deterministic results of the model. In the moderate uncontrolled pain comparison, the probabilistic INT was -£40 038. The associated 95% credible interval ranged from -£103 591 to £24 972 (includes zero) reflecting model uncertainty related to the OR of employment in those with uncontrolled moderate OA pain. For the severe uncontrolled OA pain comparison, the predicted mean probabilistic INT was -£68 924 with a 95% credible interval spanning from -£124 590 to -£4 891.

### Scenario Analyses

Base case results reflect the fiscal costs of uncontrolled OA pain using a time horizon limited to the expected work expectancy of a 50-year-old individual. This assumption was challenged by running the model over a lifetime. After 50 years, approximately 99% of the cohort is deceased. INT rose by 51% (£60 926) and 30% (£92 220) in people with uncontrolled moderate and severe OA pain compared to controlled OA pain, respectively. The increase in the absolute value of INT relates to the fact that a longer time horizon adds the costs of OA-related disability after the age of retirement to the analysis, which has proven differential between cohorts.

Still using a lifetime horizon, we ran a scenario exploring the effect of increasing SPA to 67 years. As anticipated, raising SPA to 67 years increased the difference in earnings from employment between the uncontrolled OA pain and controlled pain cohorts but increased early retirement costs. Incremental earnings became £77 519 and £135 061, a less than 1% increase from base case results for the moderate OA pain and severe OA pain comparisons. The INT decreased by 3% and 1% from base case, becoming £59 326 in people with moderate uncontrolled OA pain, and £91 181 in those with severe uncontrolled OA pain, compared to the cohort with controlled pain.

Due to the uncertainty surrounding total health-care costs, we conducted two scenarios where the incremental health-care resources associated with managing uncontrolled pain, assumed to be of 20% in those with moderate OA pain and 30% in the severe pain cohort, were increased or decreased by 10%. Unsurprisingly, reducing incremental health-care consumption by 10% resulted in INT values of £39 324 in the moderate uncontrolled OA pain comparison and £69 737 in the comparison between severe uncontrolled OA pain and controlled OA pain. In this scenario, health-care costs represented 7% and 8% of total government costs for the moderate and severe OA pain comparison, respectively.

Increasing incremental health-care costs by 10% led to INT of £41 358 in the moderate OA pain comparison and £71 771 in the severe OA pain comparison. Health-care costs became 18% and 15% of total government costs in the moderate OA pain and severe OA pain comparisons, respectively.

We explored the effect of excess pain-related mortality in a scenario analysis, but INT values were virtually unchanged from base case. Finally, we challenged the assumption around the age of OA pain onset to 45 years and updated the duration of the corresponding work expectancy to 20 years. INT values increased by 14% (£45 897) for moderate uncontrolled versus controlled OA pain, and 19% (£83 912) in the severe OA pain comparison. The absolute value of the INT changed predictably according to the scenario’s assumptions. Overall, these results suggest linearity in the model mechanics and an agreement in the direction of the fiscal effect of uncontrolled OA pain. The results for the scenarios described above can be found in the **Supplemental Materials**.

## DISCUSSION

The externalities of chronic health conditions pose considerable fiscal consequences that reach beyond government’s health-care expenditure. Our model has shown that on average, a 50-year-old with uncontrolled moderate or severe OA pain of the knee or hip will have their earnings reduced by 33% to 61% (£69 000 to £126 000), respectively, for the duration of their working life. Over the same period, the government is likely to provide 89% to 145% more financial support (£30 000 to £38 000) and incur a £24 000 to £45 000 loss in tax revenue from people with uncontrolled OA pain compared to people with controlled pain. These results may even underestimate the true dimension of the burden. An Australian study reported that the incomes of people affected by ill health was 82% lower compared to those working full-time, with those out of work paying no direct taxes and receiving more public benefits.^54^

By applying a government perspective framework to assess health and investments in health care, it is possible to understand how changes in health status can influence future tax receipts for government and transfer payments. Whilst taxes and transfers are traditionally disregarded when applying a health service perspective, these do represent real costs for government. As noted in the United Kingdom, the costs to government from ill-health in working aged populations for workless benefits and lost tax revenue from people unable to work effectively, represent approximately 85% of government costs, and health sector costs are only a minority of those costs.^55^ Accordingly, it seems meaningful to apply this framework to other disease areas, so that the public economic burden of chronic conditions is put into perspective and can be interpreted alongside traditional cost-effectiveness analyses.

In health systems financed through taxation, valuing treatments whilst ignoring their impact on the public economy can be controversial on ethical and methodological grounds. For each unemployed person, the remaining workers must pay more taxes to sustain the social system, leading them to experience reduced utility. These deadweight losses imply that spending has consequences to others not captured applying utilitarian economics. This fiscal interdependence illustrates that all members of society are linked through public economics, young and old, healthy and infirm.^56^ This fiscal reliance has both immediate and intertemporal consequences for how health budgets are spent. By considering the impact of health technologies on labour participation and productivity, analyses can become inclusive of value generated in the wider economy, rather than continuously pushing for efficacy in health systems already operating under pressure.^57^ Traditionally, measuring productivity is achieved using friction costs or human capital cost estimation.^58,59^ Whilst valid, these metrics ignore individuals’ contributions to the sustainability of a country’s social welfare system or the level of social welfare consumption. These are intimately connected to government’s budgets and decision-making across economic sectors.

One of the challenges of performing fiscal analysis in health is identifying a dominant scenario that reflects the most likely patient case encountered in the real world. This analysis considered disease onset at age 50 and the retiring age of 65 as the base case. However, changes to any of these factors can marginally influence the results as observed in our sensitivity analysis. This illustrates how fiscal consequences for government are dependent on several factors such as disease severity, age at onset, and any factors that delay progression or reduce severity. In this regard, fiscal analysis in health can reflect the general trends in how health status influences government and many factors can influence the likely conclusions to be drawn.

In the process of developing this analysis we accounted for several aspects of model validation.^60^ Firstly, the model uses a structure that reflects the natural history of OA. Inputs were sourced from independent sources, mostly nationally available. Model mechanics and equations were subject to internal cross-verification by the developing team and revised by a senior health economic modeler not directly involved in the development of the model. Relative measures of effect were obtained from peer reviewed publications as result of a targeted literature search. We used deterministic and probabilistic sensitivity analysis to explore the effect of several model inputs. The likelihood of employment in people with moderate uncontrolled OA pain was the most influential parameter, as reflected in the OSA and PSA results. Ackerman and colleagues reported that uncontrolled pain leads to a 43% reduction in the likelihood of employment in people with moderate uncontrolled OA pain, and 72% in the severe cohort.^14^ The uncertainty around the OR for moderate OA pain makes intuitive sense, as it is likely to have been inferred from a more heterogeneous cohort with pain scores ranging from mild to moderate, and varying degrees of physical limitation. Nonetheless, we believe that the effect of OA on employment is real and supported by other publications.^61,62^ Wilkie and colleagues conducted interviews in 297 English working adults aged 50 to 65, who consulted primary care practitioners due to OA symptoms.^63^ The authors concluded that OA was likely to be associated with a 3-fold increased likelihood of not being able to maintain a job.^62,63^ A comparison between fiscal analysis and other burden of disease analyses is not straightforward, due to heterogeneity in the former and because they address different economic consequences of OA. A systematic review of burden of disease studies estimated that in European countries the annual direct health-care costs represented close to 20% (€1000/€5100) of total hip and knee OA burden.^8^ The studies included in the synthesis were likely to underestimate total indirect costs as they often utilized a human capital approach and measures of productivity such as presenteeism and absenteeism that fail to capture the wider public economic consequences of more permanent decreased labour force participation. Although we were not able identify a similar analysis in the United Kingdom to calibrate our fiscal model, our results suggest that health-care costs represent a small proportion of the total costs to governments, which is supported by other published evidence.^3,64^ Additionally, the results we obtained are similar in direction and intensity to an Australian analysis using a similar framework.^65^ There are limitations to our analysis. Health-care costs were estimated from a single UK publication with small sample size (n=200) that followed people with OA or low back pain from 2004 to 2009. Additionally, because resource consumption was not reported by controlled/uncontrolled pain status, we assumed an arbitrary incremental value for implementation in the model. Uncertainty around the incremental health-care costs was addressed in a deterministic sensitivity analysis. The uncertainty about the absolute health-care cost estimation was not explored as it was perceived to have a smaller impact on the incremental results of the analysis. Due to the scarcity of relative measures of fiscal outcomes for people with controlled versus uncontrolled OA pain we had to make several modelling assumptions. Firstly, we assumed that a total WOMAC score greater than 7 was a proxy for mild to moderate uncontrolled OA pain and a score greater or equal to 39 was a proxy for severe uncontrolled OA pain. This probably over represents the contribution of OA pain as a limiting factor for employment/unemployment. In addition, we assumed that people in the controlled OA pain cohort have an identical labour force participation to those without OA. We used relative risk estimates of LT sickness and disability reported by Hubertsson et al.^15^ despite these being specific to populations with knee OA only. Finally, we assumed that the impact of these relative measures were sourced from studies outside the United Kingdom. Although we assume that OA-related loss of employment is identical between countries, what is provided by each country social welfare system may well influence individuals’ attitudes toward work.^66^ These assumptions increase uncertainty in the estimation of the true fiscal burden of uncontrolled OA pain. Further research determining the impact of chronic disease on country level labour market participation and retirement could contribute to improving the accuracy of the estimates provided by this form of analysis.

For most people, retaining the ability to remain actively employed is an important metric that contributes to individual well-being and provides people with a sense of purpose. Additionally, for people who have experienced health shocks, and have been out of work due to their poor health, returning to work is symbolic of triumphing over their health condition and a return to normality. In this context, the analysis under-represents the intangible benefits of preventing disease progression in OA. Furthermore, the costs on households can have a sustained impact over many years. A study reported in Australia found that people retiring early due to back problems experience an 87% reduction in wealth compared to those employed full-time. Additionally, those retiring early possessed 92% fewer income producing assets suggesting they will face financial pressures in retirement compared to those who are employed full-time.^67^

Despite using fiscal cost parameters that are aligned with government legislation and reports,^68^ we likely underestimated the true fiscal costs of OA. Firstly, some individuals will accumulate benefits for which we have no OA specific data, for example people getting financial support due to disability may also get housing benefits. Secondly, it is likely that after a long-term period of sickness people will be less likely to return to employment, particularly at later stages in their careers. Thirdly, we do not account for short-term absences as its costs usually falls on employers, but with the government being a large employer nationwide, there are certainly direct and indirect fiscal costs implicated. Finally, we do not account for the adverse effects of painkillers, pain related mental health problems,^69^ and OA-related limitations of informal contributions provided by retired people.

## CONCLUSIONS

The results presented here demonstrate how uncontrolled health conditions such as OA attributable pain can pose significant costs for government. Importantly, we demonstrate that in a cohort of 50-year-olds health costs are significant, but small relative to the large fiscal costs, which are currently ignored in assessments of new interventions and treatments for chronic conditions. Rather, most costs are associated with early retirement, disability, LT sickness benefits, and lost tax revenue. These findings are consistent with previous UK government reports on the health of working age populations demonstrating that National Health Service spending is estimated to be only 14% of costs of poor health in this population. Furthermore, the majority of costs described in the government working paper are attributed to workless benefits and lost tax revenue from employment inactivity in this group. The results described here likely underestimate the full economic impact of OA attributable pain. Specifically, we focused our comparative assessment to hips and knees to better understand how improving outcomes may benefit government. However, the analysis described here does not include fiscal consequences associated with back and neck pain, which also represent important sources of disability.^6^ This could lead to increased costs associated with transfer payments and lost tax revenue.

### Author contributions

RM: concept development, model design, identify input parameters, model development and analysis, interpretation of results, manuscript writing, final manuscript edits; NK: model design, identify input parameters, interpretation of results, manuscript writing, final manuscript edits; MKK: supporting literature search, cost data identification, critical appraisal of results, manuscript development; CB: concept development, input to model design, identify input parameters, interpretation of results, final manuscript edits; LA: concept development, input to model design, identify input parameters, interpretation if results, final manuscript edits; SL: identify input parameters, interpretation of results, final manuscript edits; PS: concept development, input to model design, identify input parameters, interpretation of results, final manuscript edits; and MPC: concept development, model design, identify input parameters, analysis of data, interpretation of results, manuscript writing, final manuscript edits.

### Funding Source, Conflicts of Interest

The study was sponsored by Pfizer and Eli Lilly and Company. Mark Connolly, Nikos Kotsopoulous, Rui Martins and Melodi Kosaner Kließ are employees of Global Market Access Solutions, who were paid consultants to Pfizer and Eli Lilly and Company for this study and development of this manuscript. Craig Beck, Lucy Abraham, Samuel Large and Patricia Schepman are employees of Pfizer with stock and/or stock options.

## Supplementary Material

Supplementary Materials

## References

[ref-64546] World Health Organization Chronic rheumatic conditions 2020.

[ref-64547] Palazzo Clémence, Ravaud Jean François, Papelard Agathe, Ravaud Philippe, Poiraudeau Serge (2014). The burden of musculoskeletal conditions. PLoS One.

[ref-64548] Hunter David J., Schofield Deborah, Callander Emily (2014). The individual and socioeconomic impact of osteoarthritis. Nature Reviews Rheumatology.

[ref-64549] Allen Kelli D., Golightly Yvonne M. (2015). Epidemiology of osteoarthritis: state of the evidence. Curr Opin Rheumatol.

[ref-64550] Palazzo Clémence, Nguyen Christelle, Lefevre-Colau Marie Martine, Rannou François, Poiraudeau Serge (2016). Risk factors and burden of osteoarthritis. Annals of Physical and Rehabilitation Medicine.

[ref-64551] Morgan O. J., Hillstrom H. J., Ellis S. J., Golightly Y. M., Russell R., Hannan M. T., Deland J. T. III, Hillstrom R. (2019). Osteoarthritis in England: incidence trends from National Health Service hospital episode statistics. ACR Open Rheumatology.

[ref-64552] Graham J., Novosat T., Sun H.. Osteoarthritis in a large integrated health system population: 18-Year retrospective review. Presented at: ACR Conference. Virtual, 2020..

[ref-64553] Salmon J.H., Rat A.C., Sellam J., Michel M., Eschard J.P., Guillemin F., Jolly D., Fautrel B. (2016). Economic impact of lower-limb osteoarthritis worldwide: a systematic review of cost- of-illness studies. Osteoarthritis and Cartilage.

[ref-64554] Safiri Saeid, Kolahi Ali Asghar, Smith Emma, Hill Catherine, Bettampadi Deepti, Mansournia Mohammad Ali, Hoy Damian, Ashrafi-Asgarabad Ahad, Sepidarkish Mahdi, Almasi-Hashiani Amir, Collins Gary, Kaufman Jay, Qorbani Mostafa, Moradi-Lakeh Maziar, Woolf Anthony D, Guillemin Francis, March Lyn, Cross Marita (2020). Global, regional and national burden of osteoarthritis 1990-2017: a systematic analysis of the Global Burden of Disease Study 2017. Annals of the Rheumatic Diseases.

[ref-64555] Nüesch E., Dieppe P., Reichenbach S., Williams S., Iff S., Jüni P. (2011). All cause and disease specific mortality in patients with knee or hip osteoarthritis: population based cohort study. BMJ.

[ref-64556] Hawker Gillian A., Croxford Ruth, Bierman Arlene S., Harvey Paula J., Ravi Bheeshma, Stanaitis Ian, Lipscombe Lorraine L. (2014). All-cause mortality and serious cardiovascular events in people with hip and knee osteoarthritis: a population based cohort study. PLoS One.

[ref-64557] Arthritis Research UK Osteoarthtritis in general practice: Data and perspectives 2013.

[ref-64558] Puig-Junoy Jaume, Ruiz Zamora Alba (2015). Socio-economic costs of osteoarthritis: a systematic review of cost-of-illness studies. Seminars in Arthritis and Rheumatism.

[ref-64559] Ackerman Ilana N., Ademi Zanfina, Osborne Richard H., Liew Danny (2013). Comparison of health-related quality of life, work status, and health care utilization and costs according to hip and knee joint disease severity: a national Australian study. Phys Ther.

[ref-64560] Hubertsson Jenny, Petersson Ingemar F, Thorstensson Carina A, Englund Martin (2013). Risk of sick leave and disability pension in working-age women and men with knee osteoarthritis. Annals of the Rheumatic Diseases.

[ref-64561] Laires Pedro A., Canhão Helena, Rodrigues Ana M., Eusébio Mónica, Gouveia Miguel, Branco Jaime C. (2018). The impact of osteoarthritis on early exit from work: results from a population-based study. BMC Public Health.

[ref-64562] Connolly Mark P., Kotsopoulos Nikolaos, Postma Maarten J., Bhatt Aomesh (2017). The fiscal consequences attributed to changes in morbidity and mortality linked to investments in health care: a government perspective analytic framework. Value in Health.

[ref-64563] Connolly M. (2016). The Future of Health Economics.

[ref-64564] Auerbach Alan J, Gokhale Jagadeesh, Kotlikoff Laurence J (1994). Generational accounting: a meaningful way to evaluate fiscal policy. Journal of Economic Perspectives.

[ref-64565] Cardarelli Roberto, Sefton James, Kotlikoff Laurence J. (2000). Generational accounting in the UK. The Economic Journal.

[ref-64566] Kotsopoulos Nikolaos, Connolly Mark P., Dort Thibaut, Kavaliunas Andrius (2020). The fiscal consequences of public health investments in disease-modifying therapies for the treatment of multiple sclerosis in Sweden. Journal of Medical Economics.

[ref-64567] Hawker Gillian A., Wright James G., Coyte Peter C., Williams J. Ivan, Harvey Bart, Glazier Richard, Badley Elizabeth M. (2000). Differences between men and women in the rate of use of hip and knee arthroplasty. New England Journal of Medicine.

[ref-64568] Office for National Statistics INAC01 NSA: Employment, unemployment and economic inactivity by age group (not seasonally adjusted).

[ref-64569] Office for National Statistics (2017). Disability-free prevalence rates and disability-free life expectancy by method, sex, geographical area and period 2017.

[ref-64570] Office for National Statistics (2019). Employment, unemployment and economic inactivity by age group (not seasonally adjusted).

[ref-64571] Department for Work & Pensions State Pension age review, 2017:38. https://assets.publishing.service.gov.uk/government/uploads/system/uploads/attachment_data/file/630066/print-ready-state-pension-age-review-final-report.pdf.

[ref-64608] Office for National Statistics A05 NSA: Employment, unemployment and economic inactivity by age group 2020.

[ref-64572] Kleim B. D., Malviya A., Rushton S., Bardgett M., Deehan D. J. (2015). Understanding the patient-reported factors determining time taken to return to work after hip and knee arthroplasty. Knee Surgery, Sports Traumatology, Arthroscopy.

[ref-64573] Leichtenberg C S, Tilbury C, Kuijer P P F M, Verdegaal S H M, Wolterbeek R, Nelissen R G H H, Frings-Dresen M H W, Vliet Vlieland T P M (2016). Determinants of return to work 12 months after total hip and knee arthroplasty. The Annals of The Royal College of Surgeons of England.

[ref-64574] Rolfson Ola, Ström Oskar, Kärrholm Johan, Malchau Henrik, Garellick Göran (2012). Costs related to hip disease in patients eligible for total hip arthroplasty. The Journal of Arthroplasty.

[ref-64575] Sankar A., Davis A.M., Palaganas M.P., Beaton D.E., Badley E.M., Gignac M.A. (2013). Return to work and workplace activity limitations following total hip or knee replacement. Osteoarthritis Cartilage.

[ref-64576] Scott C. E. H., Turnbull G. S., MacDonald D., Breusch S. J. (2017). Activity levels and return to work following total knee arthroplasty in patients under 65 years of age. The Bone & Joint Journal.

[ref-64577] Office for National Statistics National life tables: UK 2020.

[ref-64578] Wilkie Ross, Parmar Simran Singh, Blagojevic-Bucknall Milica, Smith Diane, Thomas Martin J, Seale Bethany Jane, Mansell Gemma, Peat George (2019). Reasons why osteoarthritis predicts mortality: path analysis within a Cox proportional hazards model. RMD Open.

[ref-64579] Briggs A., Sculpher M., Claxton K. (2006). Decision modelling for health economic evaluation.

[ref-64580] Gidwani Risha, Russell Louise B. (2020). Estimating transition probabilities from published evidence: a tutorial for decision modelers. Pharmacoeconomics.

[ref-64581] Francis-Devine B. House of Commons Library. Average earnings by age and region.

[ref-64582] Organisation for Economic Co-operation and Development Taxing wages - The United Kingdom 2020.

[ref-64583] Office for National Statistics Taxes as a percentage of income and expenditure for all households, UK, financial year ending 2018.

[ref-64584] UK Government Government Digital Service. Jobseeker’s allowance (JSA).

[ref-64585] UK Government Government Digital Service. Employment and Support Allowance (ESA).

[ref-64586] UK Government Government Digital Service. Personal Independence Payment (PIP).

[ref-64587] UK Government Government Digital Service. The new State Pension.

[ref-64588] UK Government Government Digital Service. Attendance Allowance.

[ref-64589] Hart Oliver R., Uden Ruth M., McMullan James E., Ritchie Mark S., Williams Timothy D., Smith Blair H. (2015). A study of National Health Service management of chronic osteoarthritis and low back pain. Primary Health Care Research & Development.

[ref-64590] National Health Service National Schedule of NHS costs - Year 2018-19 - NHS trust and NHS foundation trusts 2020.

[ref-64609] National Health Service Drug Tariff.

[ref-64610] Curtis L.A., Burns A. Unit Costs of Health and Social Care 2019 Kent, UK2019 [176].

[ref-64611] Culliford D.J., Maskell J., Kiran A., Judge A., Javaid M.K., Cooper C., Arden N.K. (2012). The lifetime risk of total hip and knee arthroplasty: results from the UK general practice research database. Osteoarthritis and Cartilage.

[ref-64612] National Joint Registry National Joint Registry 17th annual report 2020.

[ref-64613] Vanhegan I. S., Malik A. K., Jayakumar P., Ul Islam S., Haddad F. S. (2012). A financial analysis of revision hip arthroplasty: the economic burden in relation to the national tariff. The Journal of Bone and Joint Surgery. British volume.

[ref-64614] Kallala R. F., Vanhegan I. S., Ibrahim M. S., Sarmah S., Haddad F. S. (2015). Financial analysis of revision knee surgery based on NHS tariffs and hospital costs: does it pay to provide a revision service?. The Bone & Joint Journal.

[ref-64591] HM Treasury The Green Book: Central Government guidance on appraisal and evaluation.

[ref-64592] Schofield Deborah J, Shrestha Rupendra N, Percival Richard, Passey Megan E, Kelly Simon J, Callander Emily J (2011). Economic impacts of illness in older workers: quantifying the impact of illness on income, tax revenue and government spending. BMC Public Health.

[ref-64593] Black D.C. (2008). Review of the health of Britain's working age population: Working for a healthier tomorrow.

[ref-64594] Lee Ronald D. (2007). Demographic Change, Welfare, and Intergenerational Transfers: A Global Overview.

[ref-64595] Pettitt D., Raza S., Naughton B.. (2016). The limitations of QALY: a literature review. Journal of Stem Cell Research & Therapy.

[ref-64596] Kigozi Jesse, Jowett Sue, Lewis Martyn, Barton Pelham, Coast Joanna (2017). Valuing productivity costs using the friction-cost approach: estimating friction-period estimates by occupational classifications for the U. Health Economics.

[ref-64597] van den Hout W B (2010). The value of productivity: human-capital versus friction-cost method. Annals of the Rheumatic Diseases.

[ref-64598] Eddy David M., Hollingworth William, Caro J. Jaime, Tsevat Joel, McDonald Kathryn M., Wong John B. (2012). Model transparency and validation: a report of the ISPOR-SMDM Modeling Good Research Practices Task Force-7. Medical Decision Making.

[ref-64599] Sadosky Alesia B, Bushmakin Andrew G, Cappelleri Joseph C, Lionberger David R (2010). Relationship between patient-reported disease severity in osteoarthritis and self-reported pain, function and work productivity. Arthritis Research & Therapy.

[ref-64600] Sharif Behnam, Garner Rochelle, Sanmartin Claudia, Flanagan William M., Hennessy Deirdre, Marshall Deborah A. (2016). Risk of work loss due to illness or disability in patients with osteoarthritis: a population-based cohort study. Rheumatology.

[ref-64601] Wilkie R., Phillipson C., Hay E. M., Pransky G. (2014). Anticipated significant work limitation in primary care consulters with osteoarthritis: a prospective cohort study. BMJ Open.

[ref-64602] Brendbekken R, Vaktskjold A, Harris A, Tangen T (2018). Predictors of return-to-work in patients with chronic musculoskeletal pain: A randomized clinical trial. Journal of Rehabilitation Medicine.

[ref-64603] Schofield D., Shrestha R., Cunich M., West S. (2016). Measuring labour productivity and the benefits of interventions for osteoarthritis.

[ref-64604] Feigl Andrea B., Goryakin Yevgeniy, Devaux Marion, Lerouge Aliénor, Vuik Sabine, Cecchini Michele (2019). The short-term effect of BMI, alcohol use, and related chronic conditions on labour market outcomes: a time-lag panel analysis utilizing European SHARE dataset. PLoS One.

[ref-64605] Schofield Deborah J., Shrestha Rupendra N., Percival Richard, Callander Emily J., Kelly Simon J., Passey Megan E. (2011). Early retirement and the financial assets of individuals with back problems. European Spine Journal.

[ref-64606] Hood A., Keiller A.N. (2016). A survey of the UK benefit system.

[ref-64607] Kingsbury Sarah R., Gross Hillary J., Isherwood Gina, Conaghan Philip G. (2014). Osteoarthritis in Europe: impact on health status, work productivity and use of pharmacotherapies in five European countries. Rheumatology.

