# Supplementary material for: Comparing the Fiscal Consequences of Controlled and Uncontrolled Osteoarthritis Pain Applying a UK Public Economic Perspective
Source: J Health Econ Outcomes Res. 2021 Jun 28;8(1):127–36. doi: 10.36469/001c.24629 (PMC8238511; doi:10.36469/001c.24629)
Supplement: Supplementary Materials [file jheor_2021_8_1_24629_64064.pdf]

Journal of Health Economics and Outcomes Research

Supplementary Online Materials

Martins R, Kotsopoulos N, Ließ1 MK, Beck C, Abraham L, Large S, Schepman P, Connolly MP. Comparing the fiscal consequences of controlled and uncontrolled osteoarthritis pain applying a UK public economic perspective. *JHEOR.* 2021;8(1)125-134.
doi:10.36469/jheor.2021.24629

Figure S1: Model Diagram
Table S1: PubMed Literature Search Strategy
Figure S2: CONSORT Diagram
Table S2: Health-care Costs: Unit Costs of Health Resources and Sources
Table S3: Health-care Costs: Costs of Medicines and Modelling Assumptions
Table S4: Mean Model Inputs, Probabilistic Parameters and Sources
Table S5: Incremental Results of the Scenario Analyses, Discounted at 3.5% Annually
References

This supplementary material has been provided by the authors to give readers additional information about their work.

This is an open-access article distributed under the terms of the Creative Commons Attribution 4.0 International License (CCBY-4.0). View this license's legal deed at http://creativecommons.org/licenses/by/4.0 and legal code at http://creativecommons.org/licenses/by/4.0/legalcode for more information.

Figure S1: Model Diagram

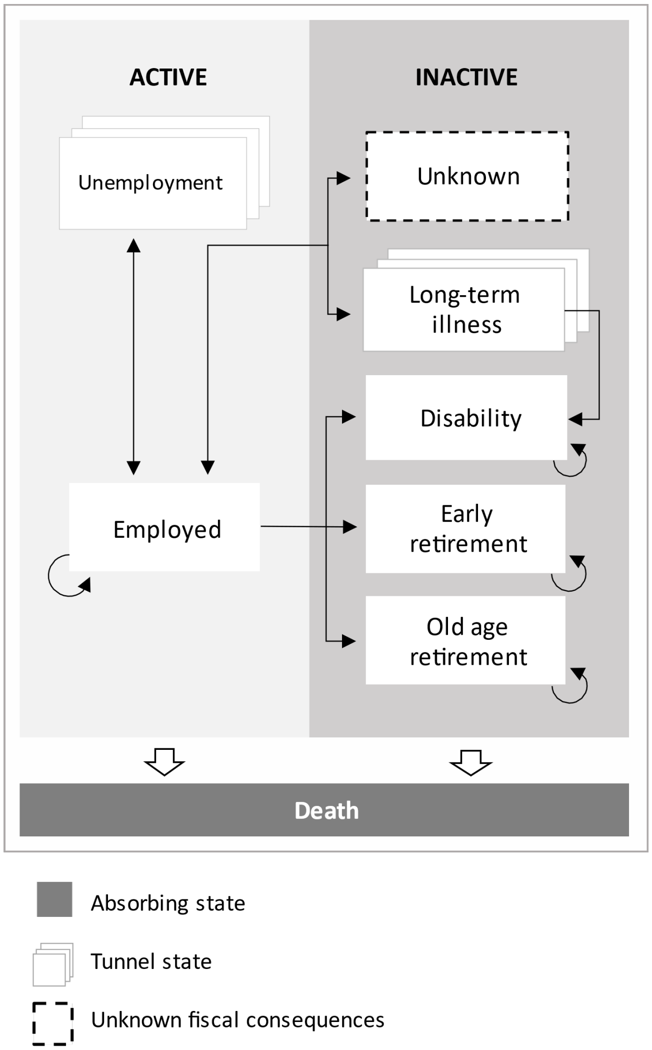

Table S1: PubMed Literature Search Strategy

#	Search terms [¥]
1	Knee osteoarthritis (MeSH)
2	Knee osteoarthritis (TiAB)
3	Gonarthro* (TiAB)
4	Gonarthritit* (TiAB)
**5**	**1 OR 2 OR 3 OR 4**
6	Hip osteoarthritis hip (MeSH)
7	Hip osteoarthritis (all)
8	Coxarthrosis (TiAB)
**9**	**6 OR 7 OR 8**
10	Osteoarthritis (MeSH)
11	(Osteoarthriti* OR OA OR osteo arthriti* or osteoarthros* or osteo arthros* or arthropath* or arthrosis or arthroses) (TiAB)
12	Degenerative arthriti* (TiAB)
13	degenerative joint disease (TiAB)
**14**	**10 OR 11 OR 12 OR 13**
15	Knee (TiAB)
16	Knee Joint (TiAB)
17	(patella* or knee ca* or kneecap* or femorotibial or femoro tibial or tibiofemoral or tibio femoral or patellofemoral or patello femoral) (TiAB)
**18**	**15 OR 16 OR 17**
**19**	**14 AND 18**
20	Hip (TiAB)
21	Hip joint (TiAB)
22	(Acetabul* OR femoral OR femoro acetabul* OR head of femur) (TiAB)
**23**	**20 OR 21 OR 22**
**24**	**14 AND 23**
25	Arthralgia (MeSH)
26	Arthralgi* (TiAB)
27	Chronic pain (MeSH)
28	Chronic pain* (TiAB)
**29**	**25 OR 26 OR 27 OR 28**
**30**	**19 AND 29**
**31**	**24 AND 29**
**32**	**5 OR 9 OR 14 OR 30 OR 31**
33	"cost of Illness"(MeSH)<br>OR "employment" (MeSH)<br>OR "productivity"(TiAB)<br>OR "lost time"(TiAB)<br>OR "employment"(TiAB)<br>OR "employment status"(TiAB)<br>OR "unemployment"(TiAB)<br>OR "underemployment"(TiAB)<br>OR "employment outcome"(TiAB)<br><br>OR "work loss"(TiAB)<br>OR "work productivity"(TiAB)<br>OR "work disability"(TiAB)<br>OR "work participation"(TiAB)<br>OR "work cessation"(TiAB)<br>OR "work status"(TiAB)<br>OR "job loss"(TiAB)<br>OR "job productivity"(TiAB)

Table S1: PubMed Literature Search Strategy

33	OR "job cessation"(TiAB)<br>OR "job status"(TiAB)<br><br>OR "workers loss"(TiAB)<br>OR "workers productivity"(TiAB)<br>OR "workers disability"(TiAB)<br>OR "workers participation"(TiAB)<br>OR "workers cessation"(TiAB)<br>OR "workers status"(TiAB)<br>OR "worker loss"(TiAB)<br>OR "worker productivity"(TiAB)<br>OR "worker disability"(TiAB)<br>OR "worker participation"(TiAB)<br>OR "worker cessation"(TiAB)<br>OR "worker status"(TiAB)<br><br>OR "workforce loss"(TiAB)<br>OR "workforce disability"(TiAB)<br>OR "workforce participation"(TiAB)<br>OR "workforce cessation"(TiAB)<br>OR "workforce status"(TiAB)<br><br>OR "vocation loss"(TiAB)<br>OR "vocation productivity"(TiAB)<br>OR "vocation disability"(TiAB)<br>OR "vocation participation"(TiAB)<br>OR "vocation cessation"(TiAB)<br>OR "vocation status"(TiAB)<br>OR "vocational loss"(TiAB)<br>OR "vocational productivity"(TiAB)<br>OR "vocational disability"(TiAB)<br>OR "vocational participation"(TiAB)<br>OR "vocational cessation"(TiAB)<br>OR "vocational status"(TiAB)<br><br>OR "occupation loss"(TiAB)<br>OR "occupation productivity"(TiAB)<br>OR "occupation disability"(TiAB)<br>OR "occupation participation"(TiAB)<br><br>OR "occupation cessation"(TiAB)<br>OR "occupation status"(TiAB)<br>OR "occupational loss"(TiAB)<br>OR "occupational productivity"(TiAB)<br>OR "occupational disability"(TiAB)<br>OR "occupational participation"(TiAB)<br>OR "occupational cessation"(TiAB)<br>OR "occupational status"(TiAB)<br>OR "employment loss"(TiAB)<br><br>OR "employment productivity"(TiAB)<br>OR "employment disability"(TiAB)<br>OR "employment participation"(TiAB)<br>OR "employment cessation"(TiAB)<br>OR "employment status"(TiAB)<br><br>OR "labour force loss"(TiAB)<br>OR "labour force productivity"(TiAB)<br>OR "labour force disability"(TiAB)<br>OR "labour force participation"(TiAB)<br>OR "labour force cessation"(TiAB)

Table S1: PubMed Literature Search Strategy

33	OR “labour force status”(TiAB)
	OR “labor force loss”(TiAB)
	OR “labor force productivity”(TiAB)
	OR “labor force disability”(TiAB)
	OR “labor force participation”(TiAB)
	OR “labor force cessation”(TiAB)
	OR “labor force status”(TiAB)
	OR “workforce loss”(TiAB)
	OR “workforce productivity”(TiAB)
	OR “workforce disability”(TiAB)
	OR “workforce participation”(TiAB)
	OR “workforce cessation”(TiAB)
	OR “workforce status”(TiAB)
	OR “work-force loss”(TiAB)
	OR “work-force productivity”(TiAB)
	OR “work-force disability”(TiAB)
	OR “work-force participation”(TiAB)
	OR “work-force cessation”(TiAB)
	OR “work-force status”(TiAB)
	OR “employee loss”(TiAB)
	OR “employee productivity”(TiAB)
	OR “employee disability”(TiAB)
	OR “employee participation”(TiAB)
	OR “employee cessation”(TiAB)
	OR “employee status”(TiAB)
	OR “employment loss”(TiAB)
	OR “employment productivity”(TiAB)
	OR “employment disability”(TiAB)
	OR “employment participation”(TiAB)
	OR “employment cessation”(TiAB)
	OR “employment status”(TiAB)
	OR “employment discontinue”(TiAB)
	OR “leaving work”(TiAB)
	OR “leaving job”(TiAB)
	OR “leaving occupation”(TiAB)
	OR “leaving employment”(TiAB)
	OR “leaving labour force”(TiAB)
	OR “leaving labor force”(TiAB)
	OR “leaving workforce”(TiAB)
	OR “leaving work-force”(TiAB)
	OR “work incapacity”(TiAB)
	OR “work incapability”(TiAB)
	OR “worker incapacity”(TiAB)
	OR “worker incapability”(TiAB)
	OR “workers incapacity”(TiAB)
	OR “workers incapability”(TiAB)
	OR “working incapacity”(TiAB)
	OR “working incapability”(TiAB)
	OR “workforce incapability”(TiAB)
	OR “workforce incapability”(TiAB)
	OR “work-force incapability”(TiAB)
	OR “work-force incapability”(TiAB)
	OR “vocational incapacity”(TiAB)

Table S1: PubMed Literature Search Strategy

33	OR "vocational incapability"(TiAB)<br>OR "occupational incapacity"(TiAB)<br>OR "occupational incapability"(TiAB)<br>OR "employment incapacity"(TiAB)<br>OR "employment incapability"(TiAB)<br>OR "employee incapacity"(TiAB)<br>OR "employee incapability"(TiAB))<br><br>OR Retirement(MeSH)<br>OR "retirement"(TiAB)<br>OR "early-retirement"(TiAB)<br>OR "early retirement"(TiAB)<br>OR "pension"(TiAB)<br>OR "early pension"(TiAB)<br>OR "disability pension"(TiAB)<br>OR "disability allowance"(TiAB)<br>OR "social insurance benefit"(TiAB)<br>OR "social insurance payment"(TiAB)<br>OR "transfer payment"(TiAB)<br><br>OR "unemployment benefits"(TiAB)<br>OR "welfare"(TiAB)<br>OR "social security"(TiAB)<br>OR "social benefits"(TiAB)
34	32 AND 33
35	Filters: published in the last 10 years; Humans; English; Adult: 19+ years

¥ Similar searches were reproduced in Embase, EconLit and CINHAL.

Figure S2: CONSORT Diagram

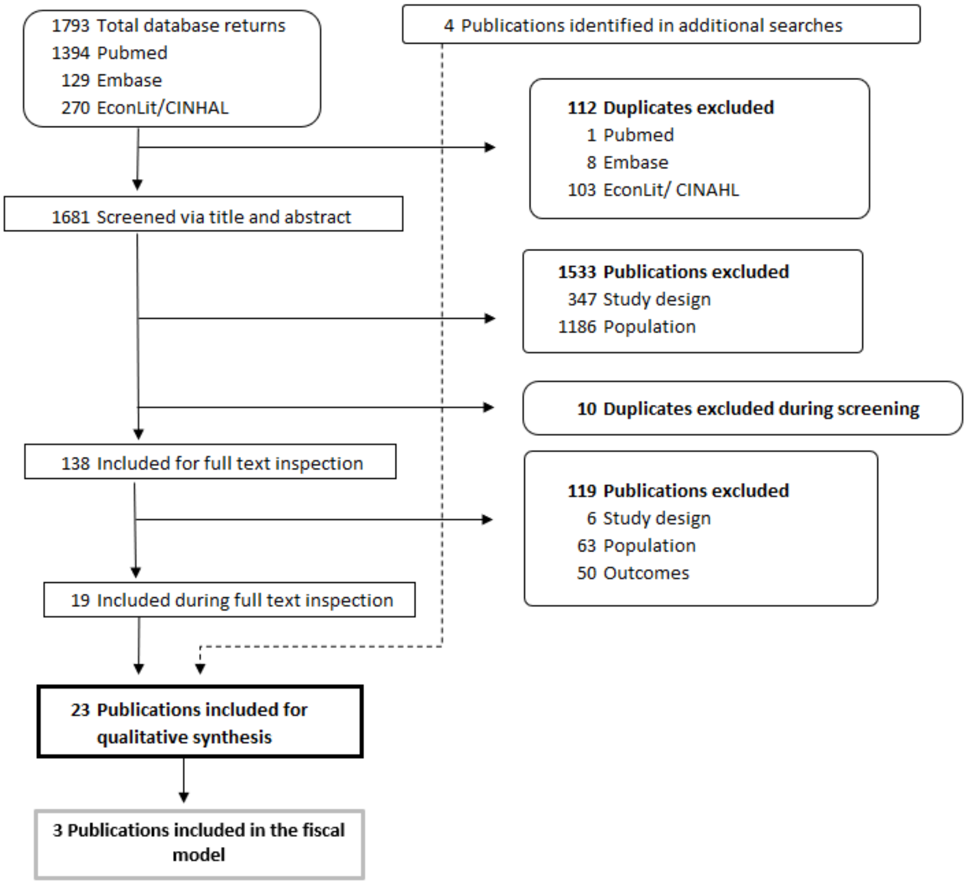

Table S2: Healthcare Costs: Unit Costs of Health Resources and Sources

Parameter	Unit Cost	Weight ¥	Source
**Referrals and Appointments**			
GP appointment	£39	-	(Curtis and Burns, 2019)
**Therapy and Investigation**			
WF01A - 650 - Physiotherapy - Non-Admitted Face-to-Face Attendance, Follow-up	£47	-	(NHS, 2020a)
IMAGOP - PF - Imaging: Outpatient - plain film	£22	-	
**Secondary Care Specialist**			
WF01A - 192 - Trauma & Orthopaedics - Non-Admitted Face-to-Face Attendance, Follow-up	£116	-	
WF01A - 191 - Pain management - Non-Admitted Face-to-Face Attendance, Follow-up	£116	-	
WF01A - 410 - Rheumatology - Non-Admitted Face-to-Face Attendance, Follow-up	£143	-	(NHS, 2020a)
**Other**			
AB23Z - 492 - Acupuncture	£67	-	(NHS, 2020a)
HN16A - 651 - Occupational therapy	£140	-	
WF01A - 190 - Anaesthetics - Non-Admitted Face-to-Face Attendance, Follow-up	£137	-	
**Outpatients**	£126		
410 - Rheumatology	£147	2 024 569	(NHS, 2020a)
101 - Urology	£108	2 007 064	
110 - Trauma & Orthopaedics	£120	5 768 964	
190 - Anaesthetics	£141	541 858	
191 - Pain Management	£157	671 381	
**Inpatient (knee)**	£1521		
HN25A - Minor Knee Procedures for Non-Trauma, 19 years and over	£2393	25	(NHS, 2020a)
HN25A - Minor Knee Procedures for Non-Trauma, 19 years and over	£1515	3517	
**Inpatient (hip)**	£1282		
HN15A - Minor Hip Procedures for Non-Trauma, 19 years and over	£2146	50	(NHS, 2020a)
HN15A - Minor Hip Procedures for Non-Trauma, 19 years and over	£1262	2109	
**A&E**			
180 - Accident & Emergency (Total outpatient attendance)	£168	-	(NHS, 2020a)
**Day Case**	£693	-	
AB18Z - Continuous Infusion of Therapeutic Substance for Pain Management	£654	10 955	(NHS, 2020a)
AB27Z - Injection of Therapeutic Substance into Joint Under Image Control for Pain Management	£716	17 959	
AB28Z - Injection of Therapeutic Substance into Joint for Pain Management	£710	5036	
**Costs of Surgery**			
**Hip Surgery**	£6714	-	
HN12A - Very Major Hip Procedures for Non-Trauma with CC Score 10+	£12 173	896	(NHS, 2020a)
HN12B - Very Major Hip Procedures for Non-Trauma with CC Score 8-9	£9517	1260	
HN12C - Very Major Hip Procedures for Non-Trauma with CC Score 6-7	£8239	2993	
HN12D - Very Major Hip Procedures for Non-Trauma with CC Score 4-5	£7271	7151	
HN12E - Very Major Hip Procedures for Non-Trauma with CC Score 2-3	£6638	16 498	
HN12F - Very Major Hip Procedures for Non-Trauma with CC Score 0-1	£6057	23 937	
**Knee Surgery**	£6296	-	
HN22A - Very Major Knee Procedures for Non-Trauma with CC Score 8+	£9675	1673	(NHS, 2020a)
HN22B - Very Major Knee Procedures for Non-Trauma with CC Score 6-7	£7661	3227	
HN22C - Very Major Knee Procedures for Non-Trauma with CC Score 4-5	£6,899	8535	
HN22D - Very Major Knee Procedures for Non-Trauma with CC Score 2-3	£6324	21 231	
HN22E - Very Major Knee Procedures for Non-Trauma with CC Score 0-1	£5699	26 470	
**Surgical Revision**			
Hip - Aseptic causes	£12 444	-	(Vanhegan et al., 2012)
Hip - Infection	£22 946	-	
Knee - Aseptic causes	£10 325	-	(Kallala et al., 2015)
Knee - Infection	£32 094	-	

¥ Used to calculate weighted averages.

Table S3: Costs of Medicines and Modelling Assumptions

Drug	Cost for Model	Units per Package	Dose per Unit	Cost per Package	Posology EMC	Developer Notes *
**Non-opioid**						
Paracetamol 500mg tablets	£48.43	32	500 mg	£1.06	2 tablets every 4 hours to a maximum of 8 tablets in 24 hours.	Assumed 8 tablets daily
Ibuprofen 400mg tablets	£68.07	24	400 mg	£1.49	Maximum of 1200 mg daily, taken after food.	Assumed 400 mg 3x daily
Diclofenacsodium 1.16% gel	£22.47	50 g	2 g	£4.61	3-4 times daily according to the need of the situation (about 2-4 g)	Assumed 3x daily (2g per application)
Celecoxib 200mg capsules	£31.19	30	200 mg	£2.56	daily dose is 200 mg taken once daily or in two divided doses	Assumed 200 mg daily
Etoricoxib 60mg tablets	£39.81	28	60 mg	£3.05	30 to 60 once daily	Assumed 200 mg daily
**Adjuvant Analgesic Drugs**						
Amitriptyline	£24.41	28	50 mg	1.87	25 mg - 75 mg daily in the evening	Assumed 50 mg daily
Pregabalin	£21.80	56	150 mg	3.34	Start with 150 mg daily, may be increased to 300 mg	Assumed 150 mg daily
**Price of Opioid and Co-prescribed Medications**						
**Strong Opioid Analgesic**						
Buprenorphine transdermal patch 10 mcg/h	£411.84	4	10 mcg/h	£31.55	Start with lowest dose (5 mcg/h), replace every 7 days	Assumed 10 mcg/h
Morphine 10 mg modified-release tablets	£126.71	60	10 mg	£5.20	One or 2 tablets twice daily	Assumed 20 mg twice daily
Fentanyl 25 mcg/h transdermal patches	£438.36	5	25 mcg/h	£17.99	Replace every 72h	Assumed 25 mcg/h
Oxycodone 10 mg modified-release tablets	£326.86	56	10 mg	£25.04	20 mg oral morphine equivalent to 10 mg oral oxycodone	Assumed 10 mg twice daily
**Weak Opioid Analgesic**						
Tramadol 100 mg modified-release tablets	£176.29	60	100 mg	£14.47	The usual initial dose is 50-100 mg tramadol hydrochloride twice daily	Assumed 100 mg twice daily
Tramadol 37.5mg / Paracetamol 325mg tablets	£119.40	60	37.7 mg/325 mg	£2.45	Do not exceed 8 daily tablets	Assumed 2x tablets, 4x daily
Codeine phosphate 15 mg tablets	£61.61	28	15 mg	£1.18	Maximum dose 240 mg/day	Assumed 15 mg, 4x daily
Dihydrocodeine 60mg modified release tablets	£67.88	56	60 mg	£5.20	One or 2 tablets twice daily	Assumed 60 mg tablet twice daily
Meptazinol 200 mg tablets	£288.61	112	200 mg	£22.11	200mg 3-6 hourly as required. Usually one tablet 4 hourly.	Assumed 200 mg 4x daily
**Compound Analgesic Containing Weak Opioid**						
Co-codamol 15mg/500mg tablets	£59.36	100	15 mg/500 mg	£4.06	200mg 3-6 hourly as required. Usually one tablet 4 hourly.	Assumed 200 mg 4x daily
**Co-prescribed Medication**						
**Laxative**						
Senna 7.5mg tablets	£14.07	60	7.5 mg	£2.31	1 or 2 tablets daily	Assumed 1 tablet daily
Docusate 100 mg capsules	£50.93	30	100 mg	£2.09	Maximum of 500 mg daily	Assumed 200 mg daily
Gastro-protective agent						
Omeprazole 20mg gastro-resistant capsules	£15.40	28	20 mg	£1.18	Once daily in the prevention of NSAID-associated gastric and duodenal ulcers	Assumed 20 mg daily
**Anti-emetic**						
Ondansetron 8mg tablets	£126.46	10	8 mg	£1.73	8 mg every 12 hours max 32 mg daily	Assumed 8 mg twice daily

Abbreviations: EMC, electronic medicines compendium; NSAID, non-steroidal anti-inflammatory drug
Medicine consumption rate was informed by the publication by Heart and colleagues (Hart et al., 2015). Prices were sourced from NHS drug tariff (NHS, 2020b). Posology was based on summary product characteristics available from the EMC (https://www.medicines.org.uk/emc/).
* Developer assumptions.

Table S4: Mean Model Inputs, Probabilistic Parameters and Sources

Parameter name	Mean	Distribution	Alpha	Beta	Source
**Baseline Probabilities of Entering Fiscal States**					
**Employment**					
35 to 49 years	0.846	Beta	5 398 272	984 310	
50 to 64 years	0.718	Beta	4 581 624	1 803 694	(ONS, 2020a)
65 to 69 years	0.107	Beta	645 836	5 414 506	
70 to 75 years	0.015	Beta	552 619	31 954 381	(ONS, 2020b)
**Unemployment**					
35 to 49 years	0.026	Beta	165 095	6 217 486	
50 to 64 years	0.028	Beta	173 692	6 211 626	(ONS, 2020a)
65 to 69 years	0.016	Beta	91 608	5 968 734	
**Long-term Sickness**					
35 to 49 years	0.031	Beta	18 616	583 073	
50 to 64 years	0.031	Beta	25 806	820 151	(ONS, 2020c)
65+ years	0.061	Beta	101 931	1 578 139	
**Disability**					
45 to 49 years	0.033	Beta	0.075	2.159	
50 to 54 years	0.039	Beta	0.105	2.615	
55 to 59 years	0.043	Beta	0.133	2.975	
60 to 64 years	0.059	Beta	0.265	4.256	
65 to 69 years	0.369	Beta	8.235	14.059	(ONS, 2019)
70 to 74 years	0.434	Beta	10.217	13.343	
75 to 79 years	0.506	Beta	12.149	11.847	
80 to 84 years	0.588	Beta	13.653	9.577	
85 to 89 years	0.705	Beta	13.962	5.855	
90+ years	0.811	Beta	11.612	2.702	
**Early Retirement**					
35 to 49 years	0.017	Beta	14 206	831 752	
50 to 64 years	0.033	Beta	56 111	1 623 960	(ONS, 2020a)
65+ years	0.115	Beta	622 429	4 794 138	Assumption
**Proportion of Males**	41%				
Males (hip)		Beta	476 754	745 692	(Morgan et al., 2019)
Males (knee)		Beta	762 097	1 010 221	
**Fiscal Outcomes (Relative measures)**					
**Employment (reduced tax)**					
Severe hip/knee vs asymptomatic [OR]	0.280	Lognormal	-1.273	0.582	(Ackerman et al., 2013)
Moderate hip/knee vs asymptomatic [OR]	0.570	Lognormal	-0.562	0.508	(Ackerman et al., 2013)
**Unemployment (job seeking benefits)**					
Hip/knee OA vs no-OA [OR]	1.970	Lognormal	0.678	0.224	(Laires et al., 2018)
**Sick Leave (not covered by employer)**					
Knee OA vs no-OA [RR] 55 to 64 years	1.800	Lognormal	0.588	0.023	
Knee OA vs no-OA [RR] 45 to 54 years	1.930	Lognormal	0.658	0.033	(Hubertsson et al., 2013)
Knee OA vs no-OA [RR] 35 to 44 years	2.160	Lognormal	0.770	0.053	
**Early Retirement**					
Hip/knee OA vs no-OA [OR]	1.430	Lognormal	0.358	0.202	(Laires et al., 2018)

Table S4: Mean Model Inputs, Probabilistic Parameters and Sources

Parameter name	Mean	Distribution	Alpha	Beta	Source
**Disability Pension**					
Knee OA vs no-OA [RR] 55 to 64 years	1.510	Lognormal	0.412	0.017	
Knee OA vs no-OA [RR] 45 to 54 years	1.610	Lognormal	0.476	0.035	(Hubertsson et al., 2013)
Knee OA vs no-OA [RR] 35 to 44 years	1.720	Lognormal	0.542	0.084	
**OA Related Mortality**					
**HR of Death per 10-point Increase in WOMAC Score**	1.040	Lognormal	0.039	0.015	(Hawker et al., 2014)
**HR of Death OA vs No OA**	1.110	Lognormal	0.104	0.025	(Wilkie et al., 2019)
**Fiscal Costs**					
**Median Weekly Pay for Full-time Employees by Age Group - Employment**					
40-49	£678	Gamma	100.000	27.100	
50-59	£623	Gamma	100.000	24.932	(Francis-Devine, 2019)
60+	£549	Gamma	100.000	21.968	
**Jobseeker's allowance - unemployment (25 or over -weekly)**	£74	Gamma	100.000	2.974	(UK Government, 2020c)
**Employment and Support Allowance (ESA) - illness (long term) - weekly**	£94	Gamma	100.000	3.758	(UK Government, 2020b)
Personal Independence Payment – Disability[¥]					
Daily living part (weekly minimum)	£60	Gamma	100.000	2.388	
Daily living part (weekly maximum)	£89	Gamma	100.000	3.566	
Mobility part (weekly minimum)	£24	Gamma	100.000	0.944	(UK Government, 2020e)
Mobility part (weekly maximum)	£62	Gamma	100.000	2.490	
**Early Retirement and State Pension Retirement**					
**Basic State Pension (weekly)**	£134	Gamma	100.000	5.360	(UK Government, 2020d)
**New Basic State Pension (weekly)**	£175	Gamma	100.000	7.008	
**Attendance Allowance (weekly minimum)[§]**	£60	Gamma	100.000	2.388	(UK Government, 2020a)
**Attendance Allowance (weekly maximum)**	£89	Gamma	100.000	3.566	
**Hip and Knee Rates Surgery[±]**					
**10-year Risk of Primary Total Hip Replacement (males)**					
40 years	0.0%				Assumption
50 years	0.8%	Beta	39	4838	
60 years	2.2%	Beta	73	3232	(Culliford et al., 2012)
70 years	3.5%	Beta	81	2225	
80 years	2.4%	Beta	30	1215	
**10-year risk of Primary Total Hip Replacement (females)**					
40 years	0.0%				Assumption
50 years	1.1%	Beta	51	4591	
60 years	3.5%	Beta	107	2962	(Culliford et al., 2012)
70 years	5.2%	Beta	136	2484	
80 years	3.5%	Beta	71	1955	
**10-year risk of Primary Total Knee Replacement (males)**					
40 years	0.0%				Assumption
50 years	0.6%	Beta	22	3643	
60 years	2.6%	Beta	84	3131	(Culliford et al., 2012)
70 years	4.4%	Beta	98	2137	
80 years	2.6%	Beta	35	1310	
**10-year Risk of Primary Total Knee Replacement (females)**					
40 years	0.0%				Assumption

Table S4: Mean Model Inputs, Probabilistic Parameters and Sources

Parameter name	Mean	Distribution	Alpha	Beta	Source
50 years	1.1%	Beta	51	4591	(Culliford et al., 2012)
60 years	3.1%	Beta	99	3105	
70 years	5.2%	Beta	136	2484	
80 years	3.2%	Beta	68	2047	
**Revision Rates**					
**Aseptic Revision (hip)**					
Cumulative hip revision rate (10 years)	4.6%	Beta	54 321	1 136 932	(National Joint Registry, 2020)
Annual probability of revision	0.5%	Beta	25 195	5 385 644	
**Septic Revision (hip)**					
Revisions due to infection per 1000 prosthesis-year	38.0%	Beta	215	350	
Annual probability of revision due to infection	0.0%	Beta	347	911 652	
**Aseptic Revision (knee)**					
Cumulative hip revision rate (10 years)	3.4%	Beta	39,161	1 105 891	
Annual probability of revision	0.3%	Beta	21,425	6 146 249	
**Septic Revision (knee)**					
Revisions due to infection per 1000 prosthesis-year	92.0%	Beta	649	56	
Annual probability of revision due to infection	0.1%	Beta	8,121	8 819 105	
**Healthcare Costs**					Calculated
**Total Controlled Pain Costs (annual)**	£916.17				
Total drugs	£203.35				
Total appointments	£712.82				
**Total Uncontrolled Pain Costs (annual)**	£1099.40				
Total drugs	£244.01				
Total appointments	£855.38				
**Drug use**					
**Proportion Treated by**					
Non-opioid	86.7%	Beta	229	35	(Hart et al., 2015)
Paracetamol	49.2%	Beta	130	134	
Systemic NSAID	58.0%	Beta	153	111	
Topical NSAID	31.8%	Beta	84	180	
COX-II inhibitor	8.0%	Beta	21	243	
Other non-opioid analgesic	4.5%	Beta	12	252	
Opioid	96.2%	Beta	254	10	
Compound analgesic (containing weak opioid)	73.5%	Beta	194	70	
Weak opioid analgesic	61.0%	Beta	161	103	
Strong opioid analgesic	16.7%	Beta	44	220	
Adjuvant analgesic drugs (eg: amitriptyline and pregabalin)	58.3%	Beta	154	110	
**Drug Costs**					
Non-opioid	£64.47	Gamma	100.000	2.579	(Hart et al., 2015)
Paracetamol	£48.43	Gamma	100.000	1.937	
Systemic NSAID	£68.07	Gamma	100.000	2.723	
Topical NSAID	£22.47	Gamma	100.000	0.899	
COX-II inhibitor	£35.50	Gamma	100.000	1.420	
Other non-opioid analgesic	£23.10	Gamma	100.000	0.924	
Opioid	£125.40	Gamma	100.000	5.016	
Compound analgesic (containing weak opioid)	£59.36	Gamma	100.000	2.374	

Table S4: Mean Model Inputs, Probabilistic Parameters and Sources

Parameter name	Mean	Distribution	Alpha	Beta	Source
Weak opioid analgesic	£116.42	Gamma	100.000	4.657	
Strong opioid analgesic	£94.32	Gamma	100.000	3.773	
Adjuvant analgesic drugs (eg: amitriptyline and pregabalin)	£23.10	Gamma	100.000	0.924	
Average	£13.48	Gamma	100.000	0.539	
**Proportion Treated by**					
**Opioids and Co-prescribed Medication**					
Strong opioid analgesic	16.7%	Beta	44	220	
Buprenorphine	9.8%	Beta	26	238	
Morphine	5.3%	Beta	14	250	
Fentanyl patch	3.8%	Beta	10	254	
Oxycodone	3.4%	Beta	9	255	
Weak opioid analgesic	61.0%	Beta	161	103	
Tramadol	41.7%	Beta	110	154	
Tramadol and paracetamol	4.5%	Beta	12	252	(Hart et al., 2015)
Codeine	13.6%	Beta	36	228	
Dihydrocodeine	12.9%	Beta	34	230	
Meptazinol	0.4%	Beta	1	263	
Co-prescribed medication	54.9%	Beta	145	119	
Laxative	40.9%	Beta	108	156	
Gastro-protective agent	48.5%	Beta	128	136	
Anti-emetic	11.4%	Beta	30	234	
**Drug Costs**					
**Opioids and Co-prescribed Medication**					
Strong opioid analgesic	£75.03	Gamma	100.000	3.001	
Buprenorphine	£411.84	Gamma	100.000	16.474	
Morphine	£126.71	Gamma	100.000	5.068	
Fentanyl patch	£438.36	Gamma	100.000	17.534	
Oxycodone	£326.86	Gamma	100.000	13.074	
Weak opioid analgesic	£97.12	Gamma	100.000	3.885	
Tramadol	£176.29	Gamma	100.000	7.052	
Tramadol and paracetamol	£119.40	Gamma	100.000	4.776	(Hart et al., 2015)
Codeine	£61.61	Gamma	100.000	2.465	
Dihydrocodeine	£67.88	Gamma	100.000	2.715	
Meptazinol	£288.61	Gamma	100.000	11.545	
Co-prescribed medication	£19.30	Gamma	100.000	0.772	
Laxative	£32.50	Gamma	100.000	1.300	
Gastro-protective agent	£15.40	Gamma	100.000	0.616	
Anti-emetic	£126.46	Gamma	100.000	5.059	
**Referrals for Pain Management**					
**Proportion Referred**					
No referral	29.2%	Beta	77	187	
Referral rate	70.8%	Beta	187	77	
**Therapy and Investigation**					(Hart et al., 2015)
Physiotherapy	39.0%	Beta	103	161	
Radiology	37.5%	Beta	99	165	

Table S4: Mean Model Inputs, Probabilistic Parameters and Sources

Parameter name	Mean	Distribution	Alpha	Beta	Source
Secondary care specialist					
Orthopaedics	30.3%	Beta	80	184	(Hart et al., 2015)
Pain clinic	8.0%	Beta	21	243	
Rheumatology	6.1%	Beta	16	248	
Other referral sites	42.0%	Beta	111	153	
**Costs**					
Therapy and investigation	£81.58	Gamma	100.000	3.263	(Hart et al., 2015)
Physiotherapy	£14.07	Gamma	100.000	0.563	
Radiology	£22.17	Gamma	100.000	0.887	
**Secondary Care Specialist**					
Orthopaedics	£116.07	Gamma	100.000	4.643	
Pain clinic	£116.18	Gamma	100.000	4.647	
Rheumatology	£143.49	Gamma	100.000	5.740	
Other referral sites	£114.79	Gamma	100.000	4.592	
**Appointments**					
**Proportion of Patients**					
Pain related GP visits	74.6%	Beta	197	67	(Hart et al., 2015)
Non-pain related GP visits	79.9%	Beta	211	53	
Physiotherapy	28.8%	Beta	76	188	
Outpatients	50.4%	Beta	133	131	
Inpatients	12.5%	Beta	33	231	
A&E	8.3%	Beta	22	242	
Day case	6.4%	Beta	17	247	
**Number of Appointments**					
Pain related GP visits	3	Uniform	0	21	(Hart et al., 2015)
Non-pain related GP visits	9.4	Uniform	0	43	
Physiotherapy	0.3	Uniform	0	7	
Outpatients	1	Uniform	0	7	
Inpatients	0.1	Uniform	0	2	
A&E	0.1	Uniform	0	1	
Day case	0.1	Uniform	0	3	
**Costs**					
Pain related GP visits	£117.69	Gamma	100.000	4.708	(Hart et al., 2015)
Non-pain related GP visits	£368.76	Gamma	100.000	14.750	
Physiotherapy	£14.07	Gamma	100.000	0.563	
Outpatients	£126.12	Gamma	100.000	5.045	
Inpatients	£1401.85	Gamma	100.000	56.074	
A&E	£16.83	Gamma	100.000	0.673	
Day case	£69.35	Gamma	100.000	2.774	
**Average**	£631.24	Gamma	100.000	25.250	

± Probabilities were converted to annual rates and then to annual probabilities (Briggs et al., 2006)
¥ Assumed 100% people with disability would receive the daily living part and mobility part (50% maximum, 50% minimum).
§ Assumed that 90% would get minimum and 10% would get maximum.
When variability data on costs was not available, a relative standard error of 10% was assumed for the gamma distributions.

Table S5: Incremental Results of the Scenario Analyses, Discounted at 3.5% Annually

	Earnings	Gross Tax Revenue	Job-Seeking Allowance	Employment and Support Allowance	Personal Independence Payment	Early Retirement Pension	Basic State Pension + Attendance Allowance	Health-care Costs	Total Transfers	Incremental Net Tax	Life-Years
**Base Case: OA Starts at Age 50, 15-year Time Horizon**											
Moderate OA	£69 383	£24 307	-£589	-£2279	-£7072	-£4059	£0	-£2034	-£16 034	£40 341	0.000
Severe OA	£126 384	£44 925	-£332	-£3475	-£11 724	-£7246	£0	-£3051	-£25 829	£70 754	0.000
**100% Males**											
Moderate OA	£66 599	£23 378	-£657	-£2078	-£6319	-£3711	£0	-£2020	-£14 784	£38 162	0.000
Severe OA	£128 794	£45 826	-£382	-£3409	-£11 273	-£7122	£0	-£3030	-£25 217	£71 042	0.000
**100% Females**											
Moderate OA	£69 805	£24 414	-£549	-£2380	-£7483	-£4221	£0	-£2044	-£16 677	£41 092	0.000
Severe OA	£122 815	£43 613	-£307	-£3477	-£11 880	-£7222	£0	-£3066	-£25 951	£69 564	0.000
**Lifetime Horizon**											
Moderate OA	£77 085	£24 124	-£589	-£2279	-£7072	-£4059	-£19,372	-£3430	-£36 802	£60 926	0.000
Severe OA	£134 369	£44 817	-£332	-£3475	-£11 724	-£7246	-£19,480	-£5145	-£47 403	£92 220	0.000
**Lifetime Horizon, SPA Increases to 67 Years**											
Moderate OA	£77 519	£24 532	-£595	-£2458	-£8317	-£4736	-£15,258	-£3430	-£34 794	£59 326	0.000
Severe OA	£135 061	£45 231	-£314	-£3556	-£13 404	-£8243	-£15,288	-£5145	-£45 950	£91 181	0.000
**Excess Healthcare Resources in Uncontrolled OA Pain Reduced by 10% (Base case used 20% for moderate and 30 for severe OA pain)**											
Moderate OA	£69 383	£24 307	-£589	-£2279	-£7072	-£4059	£0	-£1017	-£15 017	£39 324	0.000
Severe OA	£126 384	£44 925	-£332	-£3475	-£11 724	-£7246	£0	-£2034	-£24 812	£69 737	0.000
**Excess Healthcare Resources in Uncontrolled OA Pain Increased by 10% (Base case used 20% for moderate and 30 for severe OA pain)**											
Moderate OA	£69 383	£24 307	-£589	-£2279	-£7072	-£4059	£0	-£3051	-£17 051	£41 358	0.000
Severe OA	£126 384	£44 925	-£332	-£3475	-£11 724	-£7246	£0	-£4068	-£26 846	£71 771	0.000
**Excess OA Pain Mortality**											
Moderate OA	£69 506	£24 358	-£588	-£2275	-£7050	-£4044	£0	-£2020	-£15 976	£40 335	0.012
Severe OA	£126 450	£44 957	-£331	-£3470	-£11 695	-£7227	£0	-£3036	-£25 759	£70 716	0.012
**OA Starts at Age 45, 20-year Time Horizon**											
Moderate OA	£84 087	£28 217	-£803	-£2053	-£8268	-£4026	£0	-£2530	-£17 680	£45 897	0.000
Severe OA	£159 241	£5681	-£498	-£3119	-£14 484	-£7334	£0	-£3795	-£29 231	£83 912	0.000

Abbreviations: OA, osteoarthritis; SPA, state pension age.

REFERENCES

1. Ackerman IN, Ademi Z, Osborne RH, Liew D. Comparison of health-related quality of life, work status, and health care utilization and costs according to hip and knee joint disease severity: a national Australian study. *Phys Ther*. 2013;93(7):889-899. doi:10.2522/ptj.20120423.
2. Briggs A, Sculpher M, Claxton K. Decision modelling for health economic evaluation. Oxford University Press 2006.
3. Culliford DJ, Maskell J, Kiran A, et al. The lifetime risk of total hip and knee arthroplasty: results from the UK general practice research database. *Osteoarthritis Cartilage*. 2012;20(6):519-524. doi:10.1016/j.joca.2012.02.636.
4. Curtis LA, Burns A. Unit Costs of Health and Social Care 2019 Kent, UK2019 [176]. https://www.pssru.ac.uk/project-pages/unit-costs/unit-costs-2019. Accessed September 15, 2020.
5. Francis-Devine B. House of Commons Library. Average earnings by age and region. https://commonslibrary.parliament.uk/research-briefings/cbp-8456/. Accessed October 30, 2019.
6. Hart OR, Uden RM, McMullan JE, Ritchie MS, Williams TD, Smith BH. A study of National Health Service management of chronic osteoarthritis and low back pain. P*rim Health Care Res Dev.* 2015;16(2):157-166. doi:10.1017/S1463423614000140.
7. Hawker GA, Croxford R, Bierman AS, et al. All-cause mortality and serious cardiovascular events in people with hip and knee osteoarthritis: a population based cohort study. *PLoS One*. 2014;9(3):e91286. doi:10.1371/journal.pone.0091286.
8. Hubertsson J, Petersson IF, Thorstensson CA, Englund M. Risk of sick leave and disability pension in working-age women and men with knee osteoarthritis. *Ann Rheum Dis.* 2013;72(3):401-405. doi:10.1136/annrheumdis-2012-201472.
9. Kallala RF, Vanhegan IS, Ibrahim MS, Sarmah S, Haddad FS. Financial analysis of revision knee surgery based on NHS tariffs and hospital costs: does it pay to provide a revision service? *Bone Joint J*. 2015;97-b(2):197-201. doi:10.1302/0301-620x.97b2.33707
10. Laires PA, Canhao H, Rodrigues AM, Eusébio M, Gouveia M, Branco JC. The impact of osteoarthritis on early exit from work: results from a population-based study. *BMC Public Health*. 2018;18(1):472. doi:10.1186/s12889-018-5381-1
11. Morgan OJ, Hillstrom HJ, Ellis SJ, et al. Osteoarthritis in England: incidence trends from National Health Service hospital episode statistics. *ACR Open Rheumatol*. 2019;1(8):493-498. doi:10.1002/acr2.11071
12. National Joint Registry. National Joint Registry 17th annual report 2020. https://reports.njrcentre.org.uk/Portals/0/PDFdownloads/NJR%2017th%20Annual%20Report%202020.pdf. Accessed September 15, 2020.
13. National Health Service. National Schedule of NHS costs - Year 2018-19 - NHS trust and NHS foundation trusts 2020. https://www.england.nhs.uk/national-cost-collection/#ncc1819. Accessed September 24, 2020.
14. National Health Service. Drug Tariff. https://www.nhsbsa.nhs.uk/pharmacies-gp-practices-and-appliance-contractors/drug-tariff. Accessed September 15, 2020.
15. Office for National Statistics. Prevalence and employment 2019. Updated December 2, 2019. https://www.ons.gov.uk/peoplepopulationandcommunity/healthandsocialcare/disability/datasets/prevalenceandemployment. Accessed August 10, 2020.
16. Office for National Statistics. A05 NSA: Employment, unemployment and economic inactivity by age group 2020. https://www.ons.gov.uk/employmentandlabourmarket/peopleinwork/employmentandemployeetypes/datasets/employmentunemploymentandeconomicinactivitybyagegroupnotseasonallyadjusteda05nsa. Accessed August 10, 2020.
17. Office for National Statistics. 2020b. Employment by Age across different demographic and economic characteristics (Table 4) [Online]. https://www.ons.gov.uk/employmentandlabourmarket/peopleinwork/employmentandemployeetypes/datasets/employmentunemploymentandeconomicinactivitybyagegroupnotseasonallyadjusteda05nsa. Accessed August 10, 2020.
18. Office for National Statistics. INAC01 NSA: Economic inactivity by reason 2020.https://www.ons.gov.uk/employmentandlabourmarket/peopleinwork/employmentandemployeetypes/datasets/employmentunemploymentandeconomicinactivitybyagegroupnotseasonallyadjusteda05nsa. Accessed: August 10, 2020.
19. UK Government. Government Digital Service. Attendance Allowance. https://www.gov.uk/attendance-allowance. Accessed July 2020.
20. UK Government. Government Digital Service. Employment and Support Allowance (ESA). https://www.gov.uk/employment-support-allowance/what-youll-get. Accessed July 2020.
21. UK Government. Government Digital Service. Jobseeker's allowance (JSA). https://www.gov.uk/jobseekers-allowance. Accessed July 2020.
22. UK Government. Government Digital Service. The new State Pension. https://www.gov.uk/new-state-pension/what-youll-get. Accessed July 2020.
23. UK Government. Government Digital Service. Personal Independence Payment (PIP). https://www.gov.uk/pip. Accessed July 2020.
24. Vanhegan IS, Malik AK, Jayakumar P, Ul Islam S, Haddad FS. A financial analysis of revision hip arthroplasty: the economic burden in relation to the national tariff. *J Bone Joint Surg Br*. 2012;94 (5):619-623. doi:10.1302/0301-620x.94b5.27073.
25. Wilkie R, Parmar SS, Blagojevic-Bucknall M, et al. Reasons why osteoarthritis predicts mortality: path analysis within a Cox proportional hazards model. *RMD Open*. 2019;5(2):e001048. doi:10.1136/rmdopen-2019-001048.
